# Meaning in life and the mental health - addiction spiral: testing a unifying model

**DOI:** 10.3389/fpsyt.2026.1777424

**Published:** 2026-04-10

**Authors:** Or Gliksberg, Uri Lifshin, Yoav Ankri, Vera Skvirsky, Dvora Shmulewitz, Maor Daniel Levitin, Shaul Lev-Ran, Ariel Kor, Mario Mikulincer

**Affiliations:** 1 Israel Center for Addiction and Mental Health (ICAMH) and Department of Psychology, The Hebrew University of Jerusalem, Jerusalem, Israel; 2 Department of Psychology, Tel Aviv University, Tel Aviv-Yafo, Israel; 3 Department of Psychiatry, Tel Aviv Sourasky Medical Center, Tel Aviv-Yafo, Israel; 4 Lev Hasharon Mental Health Center, Netanya, Israel; 5 Hebrew University of Jerusalem Faculty of Medicine, Jerusalem, Israel

**Keywords:** addiction, behavioral addictions, meaning in life, mental health, substance use

## Abstract

**Introduction:**

Meaning in life (MIL) refers to the sense that one’s existence is coherent, purposeful, and significant. A growing body of research highlights the role of MIL in psychological well-being and resilience, as well as its inverse associations with psychological distress and problematic substance use. However, empirical work examining the joint and reciprocal relations between MIL, psychopathological symptoms, and a broad range of substance-related and behavioral addictive behaviors remains limited. The present research proposes a unifying model in which the presence of MIL is associated with lower psychological distress and reduced engagement in addictive behaviors, while psychopathological symptoms and addictions mutually reinforce one another in a downward spiral.

**Methods:**

Data were collected during the “Swords of Iron” war in Israel from two samples. Study 1 included young Israeli adults aged 18–26 (N = 1,084), and Study 2 included a quasi-representative sample of Israeli adults aged 18–70 (N = 2,912). Participants completed validated self-report measures assessing the presence of MIL, psychopathological symptoms (depression, anxiety, and post-traumatic stress symptoms), and problematic substance use and behavioral addictions, including alcohol, cannabis, prescription drugs, gambling, gaming, pornography, sex, internet, and social media use. Analyses included correlational, regression, and mediation models testing both direct and indirect associations among these constructs.

**Results:**

Across both studies, a higher presence of MIL was consistently associated with lower levels of psychopathological symptoms and reduced engagement in a wide range of substance-related and behavioral addictive behaviors. Mediation analyses indicated that psychopathological symptoms, particularly depression and post-traumatic stress symptoms, partially mediated the associations between MIL and addictive behaviors. Complementary analyses also supported indirect pathways in the opposite direction, in which addictive behaviors were linked to higher distress through reduced MIL, consistent with a bidirectional psychopathology–addiction downward spiral.

**Conclusions:**

The findings provide convergent evidence that MIL functions as a robust protective psychological factor across developmental stages and during a period of collective trauma. These results underscore the relevance of existential constructs for understanding comorbidity between psychological distress and addictive behaviors and highlight the potential value of incorporating meaning-oriented approaches into prevention and intervention strategies targeting addiction and mental health.

## Introduction

Meaning in life (MIL) refers to the perception that one’s existence is purposeful, coherent, and significant (1). Rooted in existential psychology, Frankl (2) described meaning as a fundamental human motivation, arguing that its absence, an “existential vacuum”, may lead to emotional distress, depression, and even addictive behaviors. Contemporary work positions MIL as a central component of resilience and adaptive coping (3), consistent with existential and salutogenic perspectives that emphasize meaning, coherence, and manageability as foundations of psychological well-being (4, 5).

A growing body of research links MIL to mental health. A recent meta-analysis demonstrated a robust negative association between MIL and psychological distress, alongside a positive association between the active search for meaning and distress (6). Similarly, Li et al. (7) found that a well-defined purpose in life was related to lower levels of depression and anxiety. Additional evidence suggests that MIL is negatively associated with emotion dysregulation and mediates links between maladaptive emotion-regulation strategies and depressive symptoms (8). MIL also appears protective in the context of post-traumatic stress symptoms (PTSSs); a meta-analysis of U.S. military personnel showed a moderate negative association between MIL and PTSS severity (9). Together, these findings underscore MIL as a protective psychological resource relevant to the prevention and treatment of emotional disorders.

The protective role of MIL extends to problematic substance use and addictive behaviors. Research has shown negative associations between MIL and problematic alcohol and drug use (10, 11), as well as with various behavioral addictions, including gaming (12), smartphone use (13), media and internet use (14, 15), gambling, and addictions to sex, shopping, and food (16). Csabonyi and Phillips (10) reported that while MIL was inversely related to problematic substance use among young adults, the search for meaning was positively associated with addictive behaviors. Consistent with the self-medication hypothesis (17), these findings suggest that reduced MIL may heighten vulnerability to problematic substance use and addictive behaviors as individuals attempt to cope with emotional distress. Additional work supports this perspective, highlighting MIL as a factor that fosters resilience and reduces harmful behaviors (18–20).

These associations are particularly relevant in emerging adulthood (18–25), a developmental period characterized by identity exploration, psychological instability, and heightened exposure to risky behaviors (21). Internal psychological resources such as MIL may therefore play a key role in supporting resilience and well-being during this stage (22). Moreover, it can prevent the emergence of psychopathology and harmful engagement in problematic substance use and addictive behaviors.

Importantly, accumulating evidence indicates that the relationship of psychopathological symptoms (e.g., anxiety, depression, and PTSS) with problematic substance use and addictive behaviors is bidirectional: psychological distress increases vulnerability to addictive behaviors, while addictive behaviors exacerbate distress (23–25). Individuals with low MIL may therefore be at heightened risk for entering such mutually reinforcing cycles. Prior work supports a potential mediating pathway, such as that of Yang et al. (26), who found that lower MIL predicted higher anxiety, which in turn increased risk for internet addiction. Interventions that enhance MIL have similarly demonstrated reductions in distress and subsequent problematic substance use (11), although empirical examinations of the paths going from MIL to psychopathological symptoms and from these symptoms to problematic substance use and addictive behaviors remain limited.

Complementing this evidence, research describes a *downward* sp*iral* in which psychopathological symptoms and addictive behaviors reinforce each other over time. Shame and guilt have been shown to slow reductions in stimulant use, while drug use predicts slower decreases in shame (23). Among individuals in recovery, guilt predicted subsequent PTSD symptoms and psychological comorbidity (27). From a bio-psycho-social perspective, addiction has been conceptualized as the endpoint of existential erosion characterized by diminished meaning, hope, and forgiveness (28). A recent meta-analysis similarly revealed reciprocal associations between gaming disorder and internalizing symptoms, suggesting that these spiral-like processes extend to behavioral addictions (24). It is important to note that the proposed *downward* sp*iral* is presented here as a conceptual framework derived from prior theory and empirical literature, and not as a temporally tested process within the present cross-sectional design.

Together, these findings highlight self-perpetuating reciprocal dynamics linking MIL, psychological distress, and problematic substance use and addictive behaviors. Whereas enhanced MIL may function as a stabilizing factor that disrupts this downward spiral, low MIL may accelerate emotional and behavioral dysregulation. Understanding these mechanisms is essential for clarifying how existential resources shape vulnerability and resilience in the context of substance abuse and addictive behaviors.

**Figure 1:**
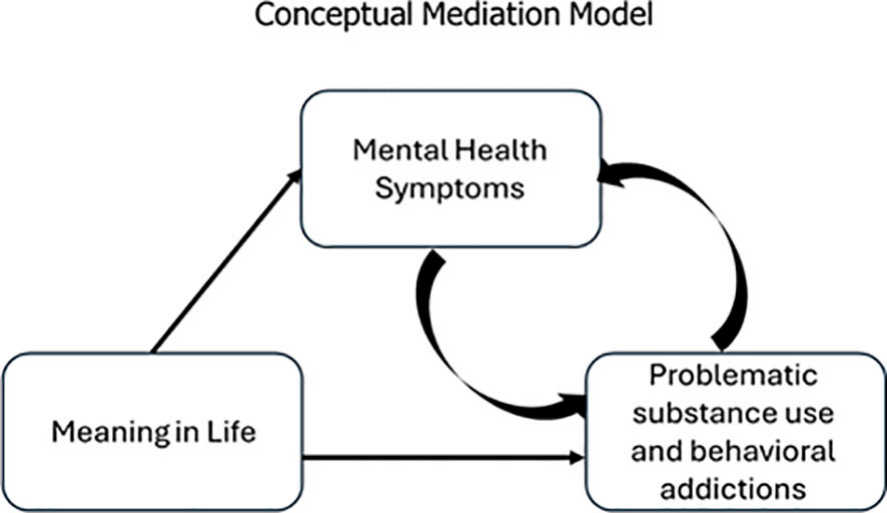
Conceptual model of the proposed associations between meaning in life, psychopathological symptoms, and addictive behaviors.

The present research aims to address this gap by examining both the direct and indirect association of MIL with mental health, problematic substance use, and addictive behaviors. We conducted two studies: Study 1 used a large sample of Israeli emerging adults (18–26; N = 1,084), and Study 2 used epidemiological data from a representative sample of Jewish adults in Israel (N = 2,912). We investigated whether MIL predicts psychopathological symptoms and problematic substance use and addictive behaviors in both Israeli young adults and adults during a period of collective trauma and war-related crisis—Hamas October 7 attack and the subsequent Swords of Iron war (29; see **Figure 1**). Specifically, we hypothesized the following:

H1. Lower MIL will be associated with increased psychopathological symptoms.
H2. Lower MIL will be associated with increased problematic substance use and addictive behaviors.
H3a. Psychopathological symptoms will mediate the association of MIL with problematic substance use and addictive behaviors.
H3b. Problematic substance use and addictive behaviors will mediate the association of MIL and psychopathological symptoms.
H4. Psychopathological symptoms and problematic substance use/addictive behaviors will be associated, consistent with a reciprocal downward spiral.

### Study 1

Study 1 aimed to examine the four hypotheses described in the general introduction within developmental and environmental contexts that heighten the risk for psychopathological symptoms, problematic substance use, and addictive behaviors. For this purpose, we approached young Israeli adults aged 18–26 (N = 1,084), who were undergoing the tumultuous developmental period of emergent adulthood, during a period of war-related crisis and collective trauma—the Hamas October 7 attack and subsequent Swords of Iron war. We asked participants to complete self-report measures tapping MIL, psychopathological symptoms (depression and anxiety), problematic substance use (alcohol, cannabis, and prescription drugs), and problematic engagement in addictive behaviors (gambling, gaming, and pornography), and we tested the four hypotheses.

## Methods

### Participants

The study was approved by the Institutional Review Board of an Israeli university. Jewish Hebrew-speaking Israelis aged 18–26, from three major regions in Israel, were recruited from a diverse panel of individuals (iPanel and panel4all) for monetary compensation (~20 ILS for a completed survey). The study was conducted from August to September 2024 during the Swords of Iron war.

After removing responses from participants with missing data or who failed manipulation checks, the final sample included 1,084 participants (422 men, 659 women, and 3 did not report gender, *M*_age_ = 23.09, *SD* = 2.27).^1^ A sensitivity analysis conducted using G*Power (30) indicated that the present sample size provided sufficient statistical power (approximately 95%) to detect very small effect sizes (f^2^ = 0.001) in hierarchical linear regression analyses. This analysis was based on two focal predictors (presence and search for MIL), a total of seven predictors including covariates, and eight outcome measures (including measures of psychopathological symptoms, problematic substance use, and addictive behaviors). To account for multiple comparisons, a conservative Bonferroni-corrected significance threshold was applied (α = .006). Importantly, all reported effects met this conservative criterion.

### Procedure and materials

After completing informed consent, participants first answered demographic questions (e.g., age, gender, religiosity, geographical district, family status, and education). Then, they received a battery of self-report questionnaires. The survey included additional questions that are not the focus of the current study and are not reported here (**Supplementary Table 1** for assessment tools administered).

MIL was measured using the 10-item MIL Questionnaire (1), which assesses the presence of MIL (five items, e.g., “My life has a clear sense of purpose”; α = .91) and the search for MIL (five items, e.g., “I am searching for meaning in my life”; α = .87). Ratings were made on a 7-point scale, ranging from 1 (*do not agree at all*) to 7 (*agree a lot*). Two scores were conducted for each participant by averaging items in each of the two subscales, with higher scores indicating a greater presence or search for MIL.

Two common psychopathological symptoms were assessed in the current study: anxiety and depression. General anxiety over the past 2 weeks was measured using the seven-item Generalized Anxiety Disorder scale (GAD-7; 31; α = .93). Depression over the past 2 weeks was assessed using the nine-item Patient Health Questionnaire-9 (PHQ-9; 32; α = .89).

Self-reported problematic substance use in the past 3 months was measured using the Alcohol, Smoking and Substance Involvement Screening Test (ASSIST 3.1; 33) for alcohol, cannabis, prescription drugs (sedatives, stimulants, and opioid painkillers), and other drugs.^2^ The ASSIST measures the frequency of non-medical use, craving, and maladaptive consequences of use. Scores were computed by summing up the items for each substance according to the ASSIST protocol.

Three behavioral addictions were assessed in the current study: gambling, addiction to pornography, and addiction to online games. First, problematic gambling was assessed using the nine-item Problem Gambling Severity Index (PGSI; 34, 35; e.g., “Have you bet more than you could really afford to lose?”; 0 = *never* to 3 = *almost always*; α = .92). Second, problematic pornography use was assessed using the six-item Problematic Pornography Consumption Scale (36; e.g., “I felt that porn is an important part of my life”; 1 = *never* to 7 = *very often\all the time*; α = .87). Third, addiction to computer and internet games was assessed using the seven-item Game Addiction Scale (37; e.g., “Played computer games to forget about your real life”; 1 = *never* to 5 = *very often*; α = .88). All measures related to the past 3 months. Scores were computed by summation.

### Transparency and openness

This study was a part of a larger survey conducted by the Israel Center for Addiction and Mental Health (ICAMH) to measure substances and behavioral addiction among Israeli youth during the Swords of Iron war. Data are available upon reasonable request.

### Statistical analysis

All analyses were conducted on IBM SPSS version 27. First, a series of zero-order Pearson’s correlations between MIL scores and measures of psychopathological symptoms, problematic substance use, and addictive behaviors were conducted. Then, hierarchical regression analyses were conducted to more closely examine the unique and joint contributions of MIL scores to the outcome variables while also examining the moderating role of gender and controlling for potential covariates. Follow-up on statistically significant interactions was performed using the *PROCESS* macro (38; version 4.2, model 1). Finally, mediational analyses were conducted using *PROCESS* (version 4.2; models 4 and 8) to examine the indirect effect of MIL scores on substance and behavioral addiction measures through psychopathological symptoms (anxiety and depression) and compare this path to alternative path models. These regression models were used to examine unique statistical associations and do not allow for causal or temporal inference. In these analyses, MIL scores (presence and search) and the mediator variables (depression and anxiety) were mean-centered at +1 and −1 *SD*. A separate analysis was conducted for each substance or behavioral addiction outcome.

## Results

As shown in **Table 1**, initial analyses indicated that the presence of MIL was related to lower levels of anxiety and depression and to lower levels of substances and behavioral addictions. In contrast, the search for MIL was slightly positively correlated with anxiety and depression, but not with any measure of problematic substance use or addictive behaviors. Also, as expected, depression and anxiety were associated with higher problematic substance use and addictive behaviors (only anxiety and problematic pornography use were not significantly associated).

**Table 1:** Descriptive statistics and Pearson’s correlations of key measures in Study 1 (N = 1,084) and Study 2 (N = 2,912).

Study 1													
	MIL presence	MIL search	Depression	Anxiety	Alcohol	Cannabis	Prescription drugs	Gambling	Gaming	Pornography			
*M*	4.91	5.21	7.91	7.27	4.56	1.09	1.55	0.29	10.11	8.65			
*SD*	1.45	1.31	6.1	5.55	5.44	4.16	4.95	1.67	5.12	5.83			
Pearson’s *r*s													
1. MIL presence	–												
2. MIL search	0.03	–											
3. Depression	−.32***	.12***	–										
4. Anxiety	−.42***	.08**	.80***	–									
5. Alcohol	−.13***	0	.15***	.19***	–								
6. Cannabis	−.12***	0.03	.09**	.06*	.18***	–							
7. Prescription Drugs	−.10***	0.05	.27***	.24***	.19***	.09**	–						
8. Gambling	−.06*	0.06	.01*	.11***	.25***	0.03	.15***	–					
9. Gaming	−.18***	0.01	.27***	.20***	.19***	-0.03	.22***	.12***	–				
10. Pornography	−.13***	-0.01	.11***	0.03	.26***	0.04	.12***	.11***	.23***	–			

Study 2													
	MIL presence	Depression	Anxiety	PCL (post-traumatic stress symptoms)	Alcohol	Cannabis	Prescription drugs	Gambling	Gaming	Pornography	Sex	Internet	Social media
*M*	5.2	4.84	3.65	14.9	5.08	1.51	2.3	0.86	10.39	4.82	3.66	23.38	11.79
*SD*	1.48	5.39	4.6	16.15	6.13	4.96	5.97	2.61	5.05	9.32	4.64	17.17	5.75
Pearson’s *r*s													
1. MILP	–												
2. Depression	−.44***	–											
3. Anxiety	−.35***	.81***	–										
4. Posttraumatic Stress Disorder	−.27***	.74***	.72***	–									
5. Alcohol	−.18***	.27***	.25***	.27***	–								
6. Cannabis	−.19***	.24***	.20***	.19***	.32***	–							
7. Prescription Drugs	−.18***	.38***	.36***	.35***	.35***	.26***	–						
8. Gambling	−.16***	.30***	.29***	.31***	.43***	.37***	.39***	–					
9. Gaming	−.22***	.39***	.37***	.42***	.33***	.30***	.31***	.40***	–				
10. Pornography	−.25***	.32***	.27***	.28***	.34***	.21***	.21***	.41***	.35***	–			
11. Sex	−.25***	.34***	.29***	.32***	.36***	.21***	.21***	.35***	.38***	.78***	–		
12. Internet	−.30***	.49***	.45***	.47***	.30***	.26***	.25***	.32***	.49***	.48***	.46***	–	
13. Social media	−.28***	.46***	.45***	.48***	.26***	.21***	.21***	.27***	.40***	.35***	.37***	.75***	–

MIL, meaning in life. PCL, Posttraumatic Stress Disorder Checklist for DSM-5 (PCL-5).

*p <.05, **p <.01, and ***p <.001.

**Figure 2:**
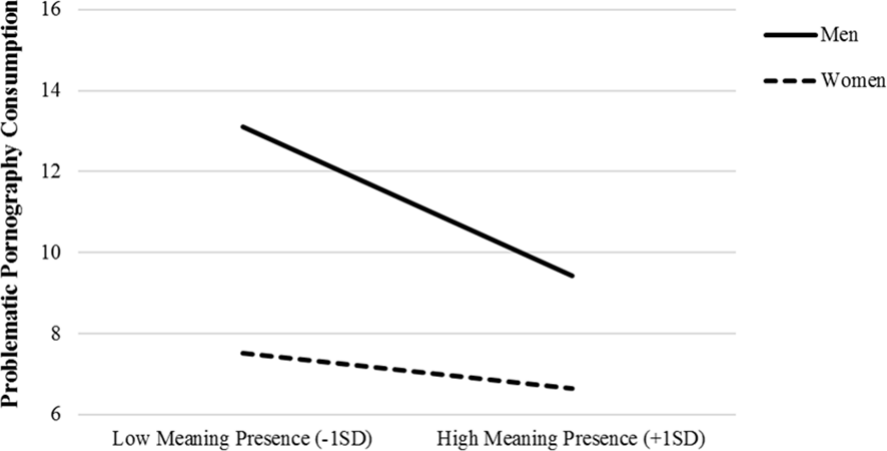
Problematic pornography consumption as a function of meaning in life (MIL) presence (mean-centered) and gender (men and women) in Study 1 (N = 1,084).

Hierarchical regression analyses indicated that the presence of MIL remained significantly associated with all outcomes after controlling for covariates (age, gender, marital status, economics, and religiosity), all *b*s > |−0.08|, *p*s <.018. Results with search of MIL were also the same when controlling for the presence of MIL and covariates; search was associated with depression and anxiety, all *b*s > |0.06|, *p*s <.005, as well as engagement in problematic gambling *b* = 0.10, *t* = 2.50, *p* = .013, but not other addiction outcomes, *b*s < |0.20|, *p*s >.087. Adding gender in the model yielded a significant interaction with the presence of MIL only for problematic pornography consumption, *b* = −0.48, *t* = 4.11, *p* <.001, Δ*R*^2^ = .013, *R*^2^ = .147. As shown in **Figure 2**, follow-up using *PROCESS* indicated that the association between the presence of MIL and problematic pornography consumption was stronger among men, *b* = −1.26, *t* = 6.91, *p* <.001, than it was among women, *b* = −0.30, *t* = 2.09, *p* = .037.

As can be seen in **Table 2**, analyses examining the mediational role of psychopathological symptoms indicated that there were statistically significant indirect effects of the presence of MIL on problematic consumption of alcohol, prescription drugs, gaming, and pornography through changes in depression, and problematic consumption of cannabis through changes in anxiety. Analyses examining the mediational role of measures tapping problematic substance use and addictive behaviors indicated that there were statistically significant indirect effects of the presence of MIL on depression through problematic consumption of alcohol, prescription drugs, gambling, and gaming. In addition, there were statistically significant indirect effects of the presence of MIL on anxiety through changes in all measures of problematic substance use and addictive behavior, aside from problematic pornography consumption.

**Table 2:** Summary of bidirectional indirect effects between mental health (MH) and problematic substance use and behavioral addictions (PSB) in Study 1 (N = 1,084).

Study 1			
Outcome	Indirect effects [95% CI]		
	Depression mediator	Anxiety mediator	PTSS mediator
Alcohol	**−0.29 [−0.46, −0.12]**	0.02 [−0.09, 0.14]	–
Cannabis	0.08 [−0.04, 0.22]	**−0.10 [−0.20, −0.01]**	–
Prescription Drugs	**−0.33 [−0.50, −0.17]**	−0.06 [−0.18, 0.05]	–
Gambling	−0.00 [−0.06, 0.07]	−0.04 [−0.09, 0.01]	–
Gaming	**−0.38 [−0.58, −0.21]**	0.03 [−0.09, 0.17]	–
Pornography	**−0.33 [−0.52, −0.14]**	**−0.20 [0.08, 0.33]**	–
Mediator	Depression outcome	Anxiety outcome	PTSS outcome
Alcohol	**−0.08 [−0.13, −0.04]**	**−0.05 [−0.09, −0.02]**	–
Cannabis	−0.01 [−0.03, 0.02]	**−0.02 [−0.05, −0.00]**	–
P. Drugs	**−0.10 [−0.17, −0.04]**	**−0.08 [−0.14, −0.03]**	–
Gambling	**−0.02 [−0.04, −0.00]**	**−0.02 [−0.05, −0.00]**	–
Gaming	**−0.15 [−0.23, −0.09]**	**−0.10 [−0.16, −0.06]**	–
Pornography	−0.03 [−0.07, 0.00]	−0.01 [−0.02, 0.04]	–

Confidence intervals are based on bootstrapped estimates (5,000 samples) using *PROCESS* Model 4. Bold effects are statistically significant (95% CI does not include zero).

MIL, meaning in life; PTSS, post-traumatic stress symptom.

Alternative path models (when the presence of MIL was the mediator or the outcome, 48 alternatives) were smaller than the ones in which the presence of MIL was the predictor (see **Table 3**). Overall, the pattern of findings of Study 1 supports the proposed theoretical framework in which MIL is centrally positioned within the addiction–mental health spiral.

**Table 3:** Strongest Alternative mediation path models in Study 1 (N = 1,084) and Study 2 (N = 2,912).

Study 1						
Original outcome	Depression (Dep)	Indirect effect [95% CI]	Anxiety (Anx)	Indirect effect [95% CI]	Post-traumatic stress (PTS)	Indirect effect [95% CI]
Alcohol	Alco. → MIL → Dep	0.06 [0.03, 0.09]	Alco. → MIL → Anx.	0.04 [0.02, 0.06]	–	–
Cannabis	Can. → MIL → Dep.	0.07 [0.04, 0.11]	Can. → MIL → Anx.	0.05 [0.03, 0.07]	–	–
P. Drugs	P. Drugs → MIL → Dep.	0.05 [0.02, 0.08]	P. Drugs → MIL → Anx.	0.03 [0.01, 0.06]	–	–
Gambling	PGSI. → MIL → Dep	0.09 [0.01, 0.23]	PGSI. → MIL → Anx.	0.06 [0.01, 0.16]	–	–
Gaming	GAS. → MIL → Dep.	0.08 [0.05, 0.12]	GAS. → MIL → Anx.	0.06 [0.04, 0.08]	–	–
Porno.	Porno. → MIL → Dep.	0.06 [0.03, 0.09]	Anx. → MIL → Porno.	0.05 [0.02, 0.07]	–	–
Study 2						
Alcohol	Alco. → MIL → Dep	0.06 [0.05, 0.08]	Alco. → MIL → Anx	0.04 [0.03, 0.05]	Alco. → MIL → PTS	0.11 [0.08, 0.14]
Cannabis	Can. → MIL → Dep	0.08 [0.07, 0.10]	Can. → MIL → Anx	0.06 [0.04, 0.07]	Can. → MIL → PTS	0.15 [0.11, 0.19]
P. Drugs	P. Drugs → MIL → Dep	0.06 [0.05, 0.08]	P. Drugs → MIL → Anx	0.04 [0.03, 0.05]	P. Drugs → MIL → PTS	0.11 [0.08, 0.13]
Gambling	PGSI. → MIL → Dep	0.13 [0.10, 0.16]	PGSI. → MIL → Anx	0.09 [0.06, 0.11]	PGSI. → MIL → PTS	0.22 [0.17, 0.29]
Gaming	GAS. → MIL → Dep	0.09 [0.07, 0.11]	GAS. → MIL → Anx	0.06 [0.04, 0.07]	GAS. → MIL → PTS	0.13 [0.10, 0.17]
Porno.	Porno. → MIL → Dep	0.06 [0.05, 0.07]	Porno. → MIL → Anx	0.04 [0.03, 0.05]	Porno. → MIL → PTS	0.09 [0.07, 0.12]
Sex	Sex. → MIL → Dep	0.11 [0.09, 0.13]	Sex. → MIL → Anx	0.07 [0.06, 0.09]	Sex. → MIL → PTS	0.18 [0.14, 0.22]
Internet	IAT. → MIL → Dep	0.03 [0.03, 0.04]	IAT. → MIL → Anx	0.02 [0.01, 0.02]	IAT. → MIL → PTS	0.04 [0.03, 0.05]
Social M.	BSMAS. → MIL → Dep	0.09 [0.07, 0.10]	BSMAS. → MIL → Anx	0.05 [0.04, 0.07]	BSMAS. → MIL → PTS	0.12 [0.09, 0.15]

MIL, meaning in life presence; Alco., problematic alcohol use (ASSIST); Can., problematic cannabis use (ASSIST); Porno., pornography; Anx., anxiety (GAD); Dep., depression (PHQ); PGSI, Problem Gambling Severity Index.

## Discussion

Results from Study 1 indicate that the presence of MIL is associated with lower levels of depression, anxiety, problematic substance use, and several behavioral addictions among emerging adults. These associations were similar across gender, aside from pornography use, for which the protective effect of MIL was stronger among men. Mediation analyses revealed that depression played a central role in explaining the links of MIL with alcohol and prescription drug misuse, as well as problematic gaming and pornography consumption. Anxiety contributed minimally, only mediating the association between MIL and problematic cannabis use. These findings suggest that MIL may reduce vulnerability to problematic behaviors primarily by mitigating depressive symptoms rather than general anxiety. However, one should take into account that although the observed pattern of regression-based associations is theoretically consistent with directional and reciprocal models linking MIL, psychological distress, and addictive behaviors, the present findings do not establish causal or temporal ordering among these constructs.

Importantly, these effects were observed only for the presence of MIL, whereas the search for meaning showed a different pattern and did not consistently relate to problematic behaviors. This aligns with literature distinguishing these constructs and suggests that actively seeking meaning reflects a more distressed psychological state.

Taken together, Study 1 highlights the salutogenic role of MIL during emerging adulthood, a period marked by instability and heightened exposure to risk. Nevertheless, given the limited representativeness of this sample, additional research is needed to assess the generalizability of these findings.

### Study 2

Study 2 was conducted to replicate and extend the results of Study 1 in a more representative sample of adult Israeli Jews. Data from the ICAMH epidemiology file (39) were used to replicate the direct and indirect contribution of the presence of MIL (we did not assess search of MIL, as this variable was found to be less associated with mental health and addiction outcomes in Study 1) to problematic substance use (alcohol, cannabis, and prescription drugs), addictive behaviors (gambling, gaming, and pornography, as well as problematic consumption of sex, internet, and social media), and psychopathological symptoms (depression, anxiety, and PTSS).

## Methods

### Participants

As detailed in Shmulewitz et al. (39), respondents were Israeli Jews aged 18–70 who completed the study online for monetary compensation (via Ipanel, for ~20 ILS). The data were collected during the Swords of Iron war in Israel (February 2025), as a second wave of a larger epidemiological study. The sample was designed to be quasi-representative, matching the prevalence of gender, age, religiosity, and area of residence to the adult Jewish population in Israel (40), allowing deviations of up to 3% from the quotas (41).

Of those invited to participate (10,869), 4,731 agreed, 339 were excluded due to quotas, and 1,460 did not complete the survey (512 failed attention checks, and 948 dropped out), and 20 were excluded based on response patterns (duplicates). The final sample included 2,912 participants (1,398 men, 1,509 women, and 5 reported “other”, *M*_age_ = 41.61, *SD* = 14.87). A sensitivity analysis conducted using G*Power (30) indicated that the present sample size provided sufficient statistical power (approximately 95%) to detect small effect sizes (f^2^ = 0.007) in hierarchical linear regression analyses. This analysis was based on one focal predictor (presence of MIL), a total of six predictors including covariates, and 12 outcome measures assessing psychopathological symptoms, problematic substance use, and behavioral addictions. To account for multiple comparisons, a conservative Bonferroni-corrected significance threshold was applied (α = .004). Importantly, all reported effects met this conservative criterion. All analyses involving gender stratification were conducted using a slightly reduced sample, as participants who did not report a defined gender category were excluded from gender-specific analyses.

### Procedure and materials

Detailed procedures and materials are available at Shmulewitz et al. (39). The procedure and materials were roughly the same as in Study 1, with various additional measures. MIL was assessed using the five-item Presence subscale of the Meaning in Life Questionnaire as in Study 1 (1). Participants did not complete the five-item Search subscale, which was not included in the survey.

All the same measures of psychopathological symptoms, problematic substance use, and addictive behaviors from Study 1 were included in the materials (all α >.89), aside from the measure of problematic pornography, which was measured with the 12-item Problematic Pornography Use Scale (42; 0 = *never true* to 5 = *almost always true*; α = .95). Additional measures of addictive behaviors included the six-item Bergen–Yale Sex Addiction Scale (BYSAS; 43; 0 = *very rarely* to 4 = *very often*, α = .88), the six-item Bergen Social Media Addiction Scale (44, 45; 1 = *very rarely* to 5 = *very often*, α = .92), and the 20-item Internet Addiction Test (46, 47; 0 = *not relevant* to 5 = *always*, α = .95). In our analysis, the Posttraumatic Stress Disorder Checklist DSM-5 version (48) was also included, consisting of 20 items, assessing PTSS symptoms due to the current war, during the past 3 months (α = .97).

### Transparency and openness

This study was not preregistered. Data were taken from a large multi-study epidemiological project (39) and are available upon reasonable request.

### Statistical analysis

All statistical analyses were the same as in Study 1. Analyses included Pearson’s correlations between MIL and all outcome measures (depression, anxiety, PTSS, and problematic consumption of alcohol, cannabis, prescription drugs, gambling, gaming, pornography, sex, internet, and social media), followed by hierarchical regressions testing unique and joint effects of MIL while controlling for covariates (age, gender, marital status, economics, and religiosity). Considering that Study 1 included only young adults, we also tested whether age moderated any of the effects. Additionally, we again examined the interaction between MIL and gender on pornography as in Study 1. Mediation analyses tested indirect effects of MIL on problematic substance use and addictive behaviors via depression, anxiety, and PTSS, with all predictors mean-centered; we ran separate models for each outcome.

## Results

As shown in **Table 1**, the presence of MIL was again significantly correlated with lower levels of anxiety, depression, and PTSS and with lower levels of problematic consumption of alcohol, cannabis, prescription drugs, gambling, gaming, pornography, sex, internet, and social media. Also, as expected, depression, anxiety, and PTSS were significantly associated with all measures of problematic substance use and addictive behaviors.

Hierarchical regression analyses indicated that the presence of MIL remained significantly associated with all outcomes after controlling for age, gender, marital status, economics, and religiosity, all *b*s > |−0.25|, *p*s <.001. Adding gender as a moderator in the model yielded a significant interaction with the presence of MIL on depression, *b* = −0.28, *t* = 2.31, *p* = .021, Δ*R*^2^ = .001, *R*^2^ = .201; PTSS, *b* = −0.77, *t* = 2.00, *p* = .046, Δ*R*^2^ = .001, *R*^2^ = .090; problematic use of cannabis, *b* = −0.60, *t* = 4.83, *p* <.001, Δ*R*^2^ = .008, *R*^2^ = .052; problematic gambling, *b* = −0.32, *t* = 5.00, *p* <.001, Δ*R*^2^ = .008, *R*^2^ = .055; problematic pornography use, *b* = −2.01, *t* = 9.73, *p* <.001, Δ*R*^2^ = .025, *R*^2^ = .223; and problematic consumption of sex, *b* = −0.79, *t* = 7.70, *p* <.001, Δ*R*^2^ = .016, *R*^2^ = .227.

**Figure 3:**
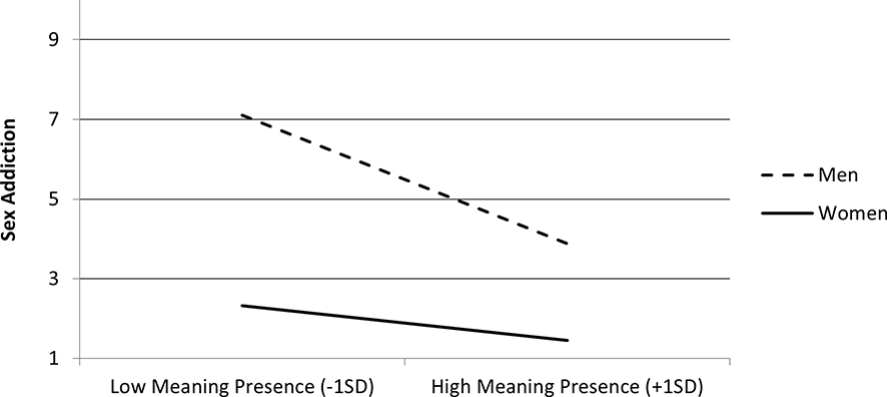
Depression scores as a function of meaning in life (MIL) presence (mean-centered) and gender (men and women) in Study 2 (N = 2,907). Figures involving gender-stratified analyses are based on a slightly reduced sample due to exclusion of participants who did not report a defined gender category.

Follow-up on the interaction on depression using *PROCESS* indicated that the negative association between the presence of MIL and depression score was slightly stronger among men, *b* = −1.76, *t* = 20.50, *p* <.001, than among women, *b* = −1.48, *t* = 17.35, *p* <.001 (see **Figure 3**).

**Figure 4:**
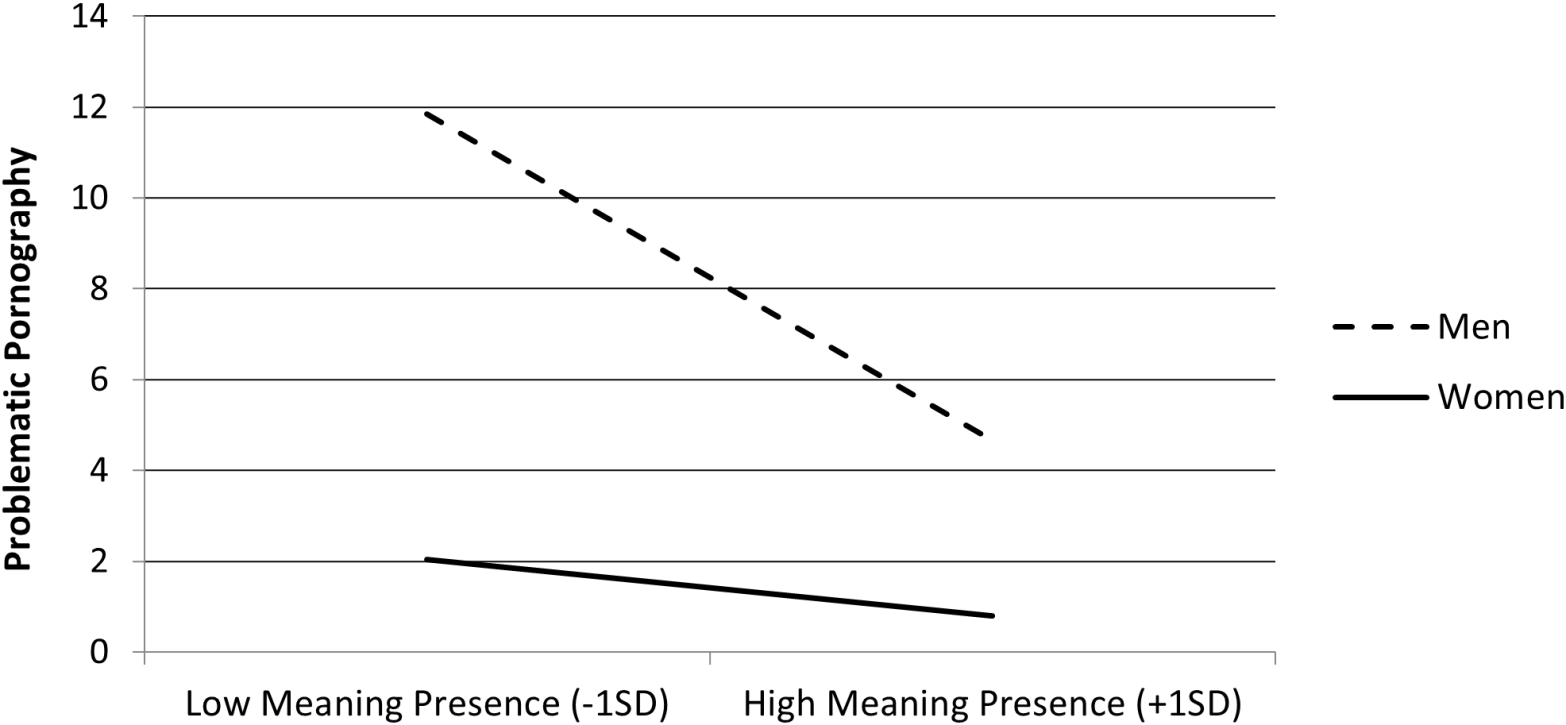
Post-traumatic stress symptoms as a function of meaning in life (MIL) presence (mean-centered) and gender (men and women) in Study 2 (N = 2,907).

Follow-up on the interaction on PTSS indicated that the negative association between the presence of MIL and PTSS Score was stronger among men, *b* = −3.46, *t* = 12.57, *p* <.001, than among women, *b* = −2.68, *t* = 9.81, *p* <.001 (see **Figure 4**).

**Figure 5:**
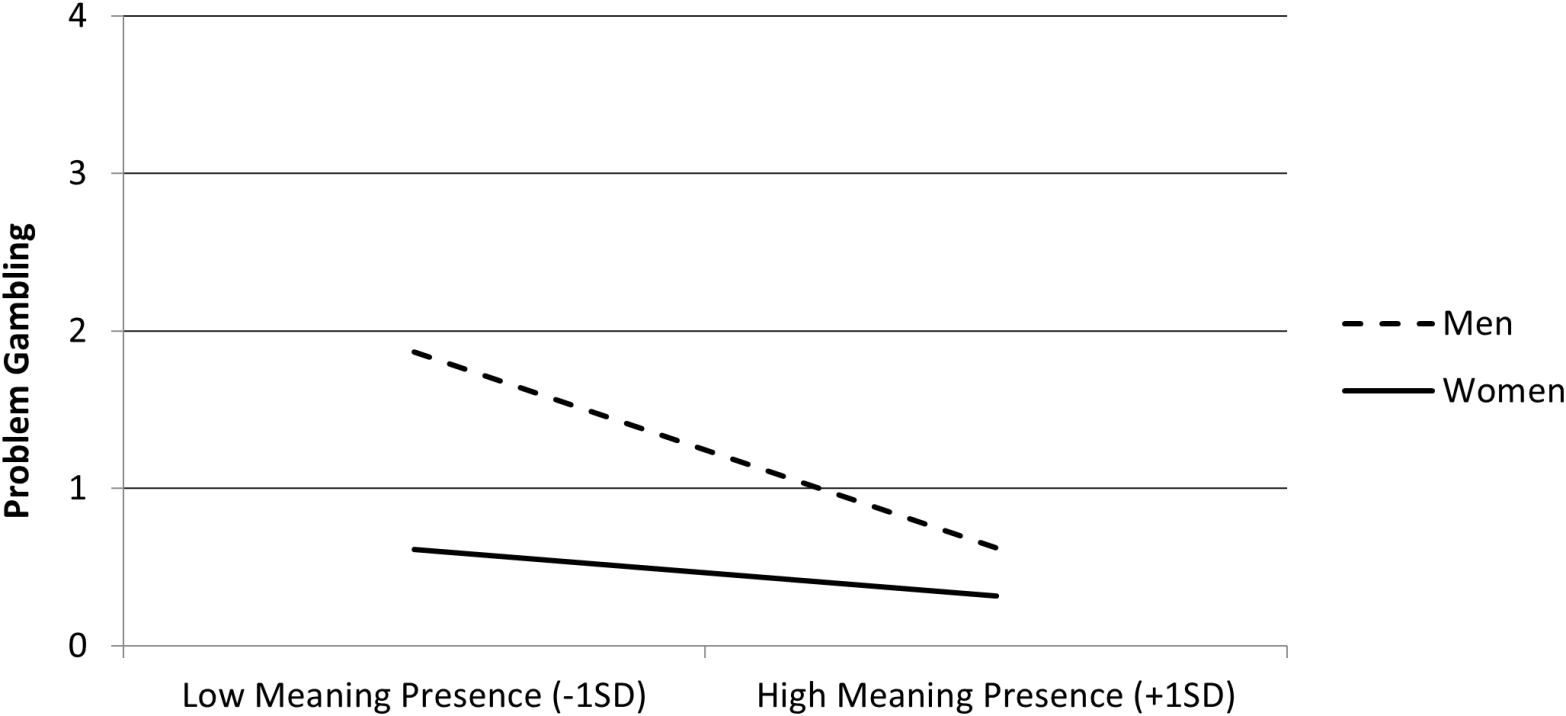
Problematic cannabis use as a function of meaning in life (MIL) presence (mean-centered) and gender (men and women) in Study 2 (N = 2,907).

Follow-up on the interaction on cannabis use indicated that the negative association between the presence of MIL and problematic cannabis use was stronger among men, *b* = −0.90, *t* = 10.47, *p* <.001, than among women, *b* = −0.32, *t* = 3.68, *p* <.001 (see **Figure 5**).

**Figure 6:**
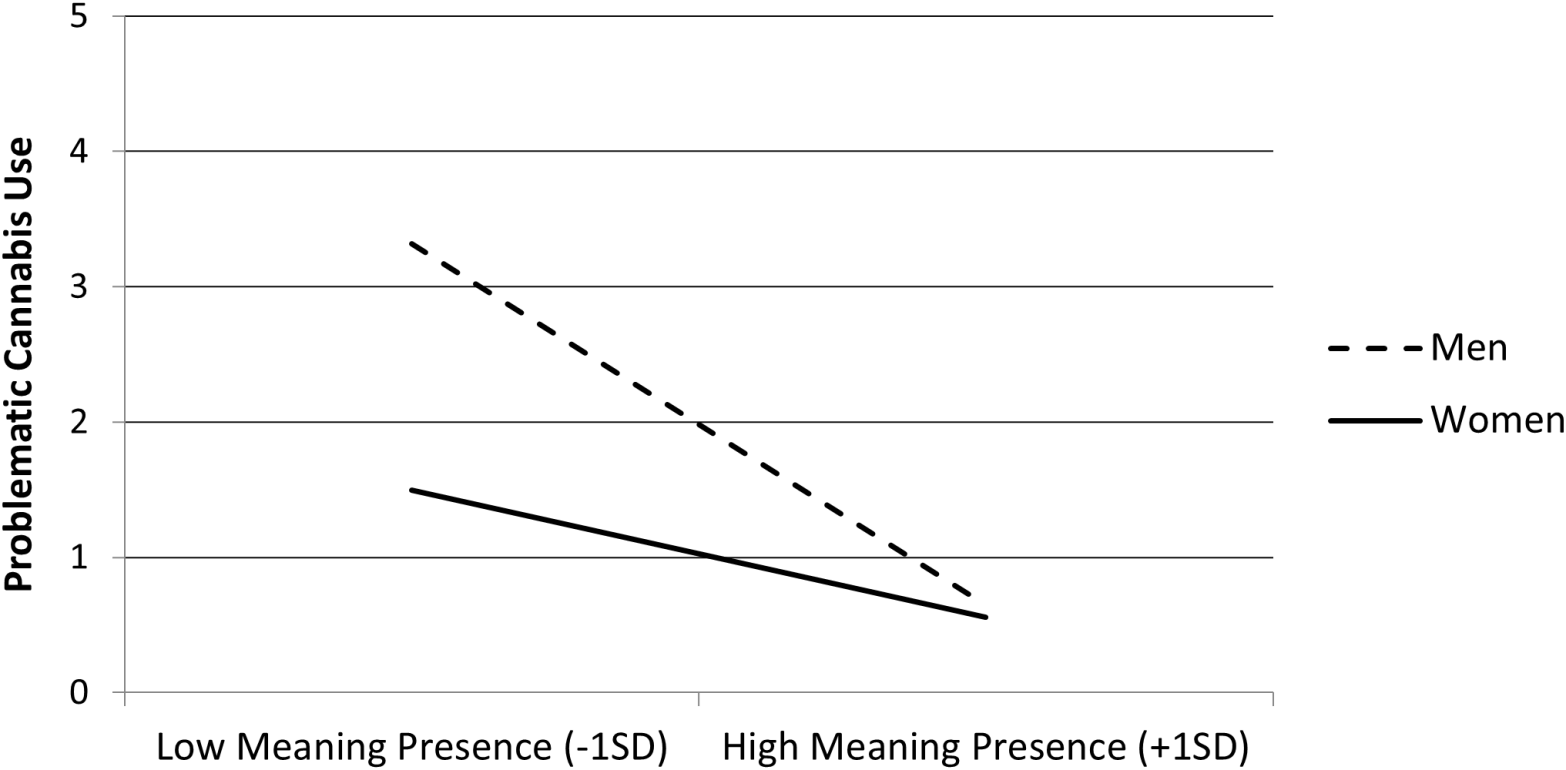
Problem gambling as a function of meaning in life (MIL) presence (mean-centered) and gender (men and women) in Study 2 (N = 2,907).

Follow-up on the interaction on gambling indicated that the negative association between the presence of MIL and problematic gambling was stronger among men, *b* = −0.42, *t* = 9.25, *p* <.001, than among women, *b* = −0.10, *t* = 2.21, *p* = .027 (see **Figure 6**).

**Figure 7:**
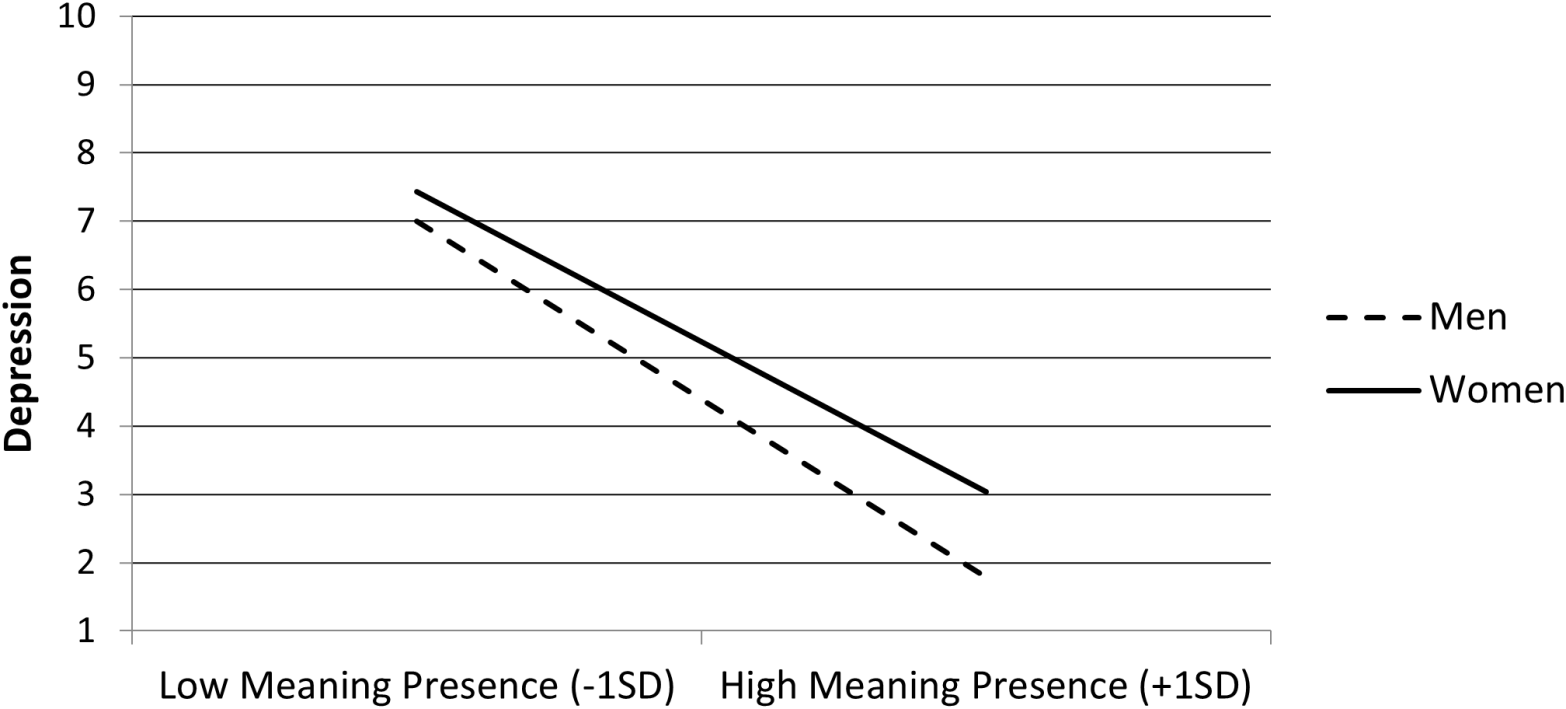
Problematic pornography consumption as a function of meaning in life (MIL) presence (mean-centered) and gender (men and women) in Study 2 (N = 2,907).

Follow-up on the interaction on pornography indicated that the negative association between the presence of MIL and problematic pornography was stronger among men, *b* = −2.43, *t* = 16.57, *p* <.001, than among women, *b* = −0.42, *t* = 2.88, *p* = .004 (see **Figure 7**).

**Figure 8:**
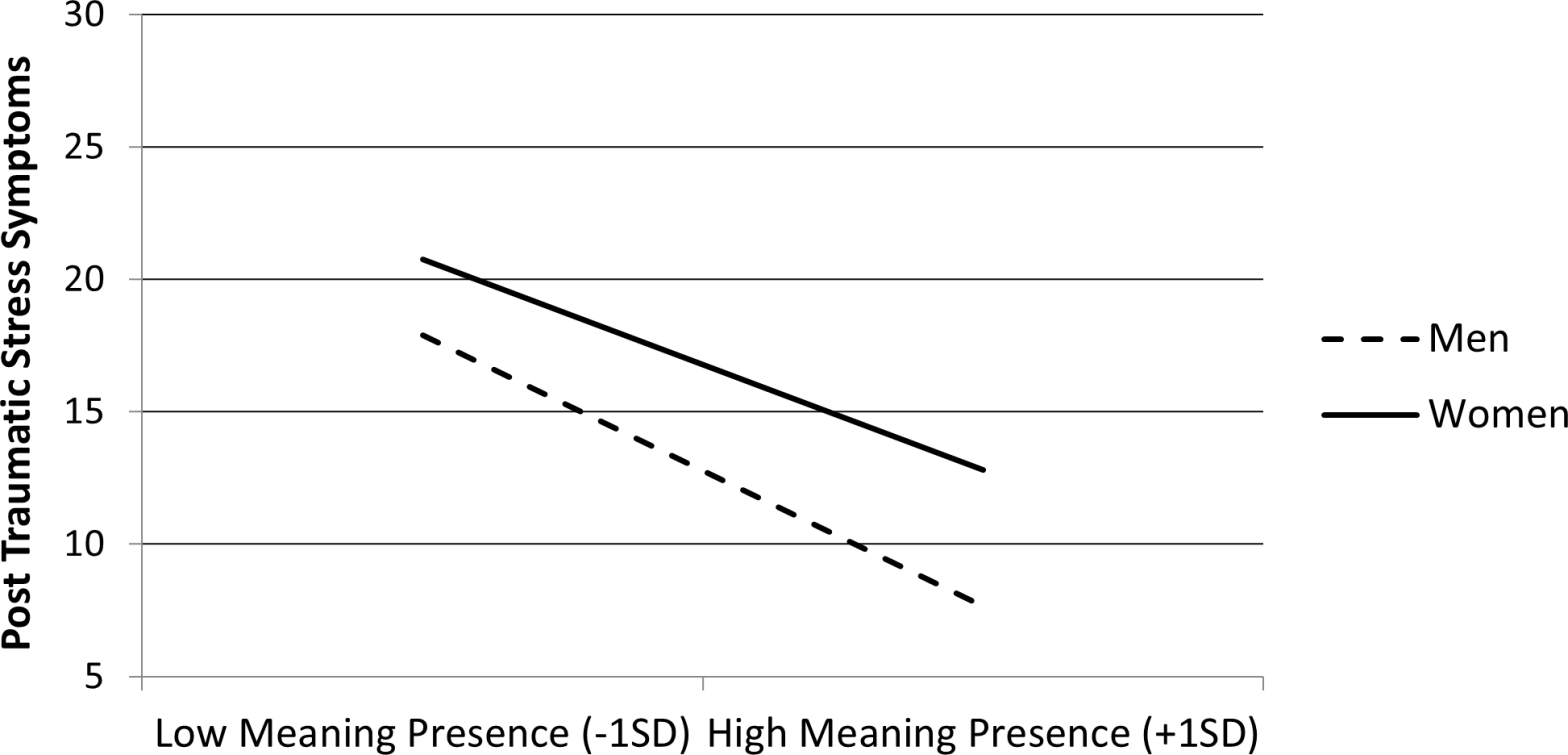
Sex addiction as a function of meaning in life (MIL) presence (mean-centered) and gender (men and women) in Study 2 (N = 2,907).

Follow-up on the interaction on sex addiction indicated that the negative association between the presence of MIL and problematic consumption of sex was stronger among men, *b* = −1.09, *t* = 14.89, *p* <.001, than among women, *b* = −0.29, *t* = 4.06, *p* <.001 (see **Figure 8**).

We conducted separate moderation analyses for each dependent variable with age as the moderator. Significant interactions with the presence of MIL emerged only on problematic consumption of alcohol (*b* = −0.01, *t* = 2.06, *p* = .040, Δ*R*^2^ = .001, *R*^2^ = .033) and sex (*b* = −0.01, *t* = 3.16, *p* = .002, Δ*R*^2^ = .003, *R*^2^ = .069). Interactions were non-significant on all other dependent variables, all *F* > 2.96, *p*s >.086. Follow-up on the statistically significant interactions using *PROCESS* indicated that the association between the presence of MIL and problematic consumption of alcohol was stronger among older participants (*b* = −0.89, *t* = 7.44, *p* <.001) than among younger participants (*b* = −0.56, *t* = 5.47, *p* <.001). Similarly, the association between the presence of MIL and problematic consumption of sex was stronger among older participants (*b* = −0.95, *t* = 10.68, *p* <.001) than among younger participants (*b* = −0.57, *t* = 7.52, *p* <.001).

Finally, an analysis examining the mediational role of depression, anxiety, and PTSS revealed the following patterns of mediation (see **Table 4**). First, there were statistically significant indirect effects of the presence of MIL through depression on all outcomes aside from problem gambling. Second, there were statistically significant indirect effects of the presence of MIL through PTSS on all outcomes aside from problematic use of cannabis. Third, there were statistically significant indirect effects of the presence of MIL through anxiety only on problematic consumption of prescription drugs, internet, and social media. Analyses examining the mediational role of addiction measures indicated that there were statistically significant indirect effects of the presence of MIL on depression, anxiety, and PTSS through all measures of problematic substance use and addictive behaviors.

**Table 4:** Summary of bidirectional indirect effects between mental health (MH) and problematic substance use and behavioral addictions (PSB) in Study 2 (N = 2,912).

Study 2			
Outcome	Indirect effects [95% CI]		
	Depression mediator	Anxiety mediator	PTSS mediator
Alcohol	**−0.15 [−0.29, −0.02]**	−0.08 [−0.19, 0.02]	**−0.16 [−0.23, −0.09]**
Cannabis	**−0.20 [−0.34, −0.07]**	−0.03 [−0.12, 0.06]	−0.04 [−0.11, 0.03]
P. Drugs	**−0.36 [−0.52, −0.21]**	**−0.12 [−0.25, 0.00]**	**−0.15 [−0.23, −0.07]**
Gambling	−0.06 [−0.12, 0.00]	−0.05 [−0.11, 0.00]	**−0.09 [−0.13, −0.05]**
Gaming	**−0.16 [−0.28, −0.04]**	0.07 [−0.16, 0.02]	**−0.25 [−0.33, −0.19]**
Pornography	**−0.45 [−0.68, −0.22]**	−0.01 [−0.18, 0.16]	**−0.19 [−0.32, −0.06]**
Sex	**−0.22 [−0.33, −0.11]**	0.00 [−0.08, 0.08]	**−0.14 [−0.20, −0.08]**
Internet	**−0.99 [−1.36, −0.64]**	**−0.32 [−0.58, −0.05]**	**−0.75 [−0.97, −0.55]**
Social media	**−0.15 [−0.27, −0.04]**	**−0.16 [−0.25, −0.07]**	**−0.31 [−0.39, −0.24]**
Mediator	Depression outcome	Anxiety outcome	PTSS outcome
Alcohol	**−0.13 [−0.17, −0.09]**	**−0.11[−0.14, −0.08]**	**−0.43 [−0.56, −0.32]**
Cannabis	**−0.11 [−0.15, −0.07]**	**−0.08 [−0.12, −0.05]**	**−0.30 [−0.42, −0.19]**
P. Drugs	**−0.21[−0.26, −0.15]**	**−0.17 [−0.22, −0.13]**	**−0.62 [−0.77, −0.46]**
Gambling	**−0.13[−0.18, −0.09]**	**−0.12 [−0.16, −0.08]**	**−0.47 [−0.62, −0.33]**
Gaming	**−0.25 [−0.31, −0.20]**	**−0.21 [−0.27, −0.19]**	**−0.91 [−1.10, −0.73]**
Pornography	**−0.20 [−0.26, −0.15]**	**−0.15 [−0.20, −0.11]**	**−0.61 [−0.79, −0.46]**
Sex	**−0.22 [−0.27, −0.17]**	**−0.17 [−0.22, −0.13]**	**−0.71 [−0.89, −0.55]**
Internet	**−0.44 [−0.50, −0.37]**	**−0.36 [−0.42, −0.30]**	**−1.43 [−1.65, −1.22]**
Social media	**−0.37 [−0.44, −0.31]**	**−0.33 [−0.39, −0.28]**	**−1.34 [−1.57, −1.13]**

Confidence intervals are based on bootstrapped estimates (5,000 samples) using *PROCESS* Model 4. Bold effects are statistically significant (95% CI does not include zero).

MIL, meaning in life; PTSS, post-traumatic stress symptom.

Alternative path models (when the presence of MIL was the mediator or the outcome, 108 alternative models) were smaller than the ones in which the presence of MIL was the predictor (see **Table 3**). This replication and extension are consistent with the proposed theoretical framework illustrated in **Figure 1**, which presents a conceptual model of the relationships between MIL, psychopathological symptoms, and addictive behaviors.

## Discussion

Study 2 replicated and expanded the findings of Study 1 within a large, quasi-representative adult sample. As expected, the presence of MIL was inversely associated with problematic use of alcohol, cannabis, and prescription drugs as well as a broad spectrum of addictive behaviors, including problematic consumption of gambling, gaming, pornography, sex, internet, and social media. Mediation analyses showed that depression and PTSS were the most robust and consistent mediators linking MIL to measures of substance and behavioral addiction across domains. These emotional difficulties accounted for a substantial portion of the protective association of MIL, suggesting that meaning exerts its effects largely by reducing depressive symptoms and trauma-related distress. In contrast, anxiety played a more selective and modest mediating role, emerging only for a small number of outcomes. This pattern underscores the relative importance of depression and PTSS in the mechanisms through which MIL protects against addictive behaviors. The replication of results in a diverse adult population, during a period of societal crisis and collective trauma, highlights the generalizability of the protective function of MIL beyond emerging adulthood and into later developmental stages.

### General discussion

The present research examined the direct and indirect associations between MIL, psychopathological symptoms, and substance and behavioral addictions across two Israeli samples during a period of war-related crisis. Across both studies, regression-based analyses consistently showed that MIL was independently associated with lower levels of psychopathological symptoms and lower engagement in a broad range of addictive behaviors. Although the observed pattern of associations is theoretically consistent with reciprocal and spiral-like models linking psychological distress and addictive behaviors, the present findings do not establish temporal or reciprocal processes. Rather, they support the conceptual plausibility of such models based on cross-sectional, regression-based evidence.

The current findings suggest that MIL may function as a stable protective resource across both young and older adulthood. These findings extend previous work (6, 9, 19) by showing that MIL’s adaptive role is not only limited to emotional functioning but also spans a spectrum of maladaptive behaviors, including both problematic substance use and behavioral addictions, even under conditions of collective trauma. A notable pattern across both studies was the central role of depression and PTSS, which emerged as the most consistent mediators linking MIL with problematic substance use and addictive behaviors. Anxiety played a weaker and more selective role, suggesting that MIL may exert stronger influence on mood-related and trauma-related processes than on generalized hyperarousal or fear-based symptoms (49, 50). Developmental factors may also contribute to these differences, as older adults often rely more heavily on meaning-making strategies when coping with trauma and uncertainty (26, 51).

In both studies, higher MIL was associated with lower involvement in several behavioral addictions, most consistently gaming and pornography use, and in the adult sample, also problematic internet and social media use. In contrast, associations with problematic substance use were more variable, emerging reliably only in the adult sample. This discrepancy may reflect developmental differences: whereas young adults often engage in substance use for social or exploratory reasons that are less tied to distress or meaning making, substance use among older adults more often serves an emotion-regulation function. As adults typically possess more established sources of meaning and purpose, MIL may play a stronger protective role against substance-related behaviors in this life stage. From the perspective of the self-medication hypothesis (17), individuals with higher MIL may have less need to use substances or engage in addictive behaviors as a means for regulating distress.

The findings also resonate with theoretical models describing bidirectional and self-reinforcing dynamics between psychological distress and addictive behaviors. Prior research suggests that distress can increase vulnerability to addictive behaviors, while engagement in such behaviors exacerbates distress over time, creating a downward spiral of emotional and behavioral dysregulation. The present results provide strong empirical support for this perspective.

Across both studies, not only did lower MIL was associated with higher psychopathological symptoms, which, in turn, was associated with more severe problematic substance use and addictive behaviors, but the alternative model demonstrated the complementary direction: higher levels of addictive behaviors were associated with lower MIL, which subsequently related to higher levels of depression and anxiety (and PTSS in Study 2). This bidirectional pattern, in which distress increases vulnerability to addictive behaviors, and addictive behaviors further erode MIL and intensify distress, reflects a self-perpetuating cycle consistent with downward spiral models of psychopathology and addiction (52). In this framework, MIL appears to function as a stabilizing psychological resource that may help interrupt or buffer against these recursive cycles.

### Implications for prevention and intervention

These findings have important implications for prevention and intervention. Meaning-centered interventions (11, 53, 54) may strengthen resilience and reduce vulnerability to addictive behaviors by fostering coherent life narratives, enhancing emotional regulation, and providing adaptive alternatives for coping with stress. Evidence from clinical and non-clinical contexts shows that such interventions reduce depressive and anxiety symptoms, improve quality of life, and decrease engagement in maladaptive behaviors. Integrating meaning-based modules into existing addiction and mental health treatments, while tailoring them to developmental stage, trauma exposure, and cultural context, may further enhance their efficacy. Future longitudinal work should evaluate whether strengthening MIL can effectively counteract or reverse downward spirals of distress and addictive behavior.

### Limitations and future directions

Several limitations should be noted. The cross-sectional design precludes causal inference (55), and future longitudinal and experimental studies are needed to establish directional pathways. Because the data are cross-sectional, mediation analyses cannot be used to test temporal or reciprocal processes implied by the downward spiral model, which should therefore be interpreted as a conceptual framework rather than an empirically established process.

Across both studies, sensitivity analyses indicated that the large sample sizes provided high statistical power to detect small effects in hierarchical regression models, even under conservative significance thresholds. To reduce the risk of Type I error, given the number of outcomes examined, conservative corrections for multiple comparisons were applied in both studies, and all reported effects met these criteria (all *p*-values <.001, except for the main effect of problematic gambling in Study 1, but also this was under *p* <.001 among men). At the same time, given the high statistical power and the exclusive reliance on self-report measures, statistically significant associations should be interpreted with caution. Accordingly, the emphasis of the present findings is placed on the consistency and convergence of observed patterns across outcomes and samples rather than on the magnitude or practical significance of individual effects. Future research should employ longitudinal and clinical designs to examine the temporal dynamics implied by the proposed model and to assess the practical and clinical significance of MIL in the context of addiction and mental health.

Although the samples were large and quasi-representative, they were internet-based and may not fully reflect the broader Israeli population (56). Additionally, the present findings were derived from non-clinical samples (57); replication in clinical and high-risk groups is necessary to examine generalizability and applied relevance.

## Conclusion

Using regression-based models, these studies demonstrated that MIL was uniquely associated with key mental health and addiction-related outcomes, in patterns consistent with theoretical models of mental health-addiction dynamics. While the findings are consistent with theoretically proposed directional models, they should be interpreted as reflecting regression-based associations rather than causal processes.

The consistent mediating roles of depression and PTSS, coupled with the bidirectional effects observed in the alternative path models, support a conceptualization of MIL as a critical existential resource capable of interrupting downward spirals linking distress and addiction. These findings underscore the importance of incorporating meaning-centered principles into prevention and intervention strategies targeting mental health and addiction.

## Data Availability

The raw data supporting the conclusions of this article will be made available by the authors, without undue reservation.

## Footnotes

1 In Study 1, 2,117 agreed to participate, 938 did not complete the survey, 90 failed attention checks, and 5 were excluded based on response patterns or duplicates.

2 We used the prescription drug category by computing the max scores of stimulants, sedatives, and opioids, considering that there were low rates of each in the sample.
