# Supplementary material for: Meaning in life and the mental health - addiction spiral: testing a unifying model
Source: Front Psychiatry. 2026 Apr 10;17:1777424. doi: 10.3389/fpsyt.2026.1777424 (PMC13106114; doi:10.3389/fpsyt.2026.1777424)
Supplement: Supplementary file 1 [file DataSheet1.docx]

**Supplementary material**

**Table S1**

*Assessment Tools Administered in Study 1*

| Lafontaine, M. F., Brassard, A., Lussier, Y., Valois, P., Shaver, P. R., & Johnson, S. M. (2016). Selecting the best items for a short-form of the Experiences in Close Relationships questionnaire. *European Journal of Psychological Assessment, 32*, 140-154. | ECR-12 |
| --- | --- |
| Fraley, R. C., & Davis, K. E. (1997). Attachment formation and transfer in young adults’ close friendships and romantic relationships. *Personal relationships, 4*(2), 131-144.‏ | WHOTO |
| Steger, M. F., Frazier, P., Oishi, S., & Kaler, M. (2006). The Meaning in Life Questionnaire: Assessing the presence of and search for meaning in life*. Journal of Counseling Psychology, 53*(1), 80-93. https://doi.org/10.1037/0022-0167.53.1.80 | MIL |
| Campbell‐Sills, L., & Stein, M. B. (2007). Psychometric analysis and refinement of the Connor–Davidson Resilience Scale (CD‐RISC): Validation of a 10‐item measure of resilience. *Journal of Traumatic Stress, 20*(6), 1019-1028.‏ | CD-RISC |
| Fraley, R. C., Heffernan, M. E., Vicary, A. M., & Brumbaugh, C. C. (2011). The Experiences in Close Relationships-Relationship Structures questionnaire: A method for assessing attachment orientations across relationships. *Psychological Assessment, 23*, 615-625. | ECR-RS |
| Kroenke, K., Spitzer, R. L., Williams, J. B., Monahan, P. O., & Löwe, B. (2007). Anxiety disorders in primary care: Prevalence, impairment, comorbidity, and detection. *Annals of internal medicine, 146(*5), 317-325. | GAD-2 |
| Löwe, B., Kroenke, K., & Gräfe, K. (2005). Detecting and monitoring depression with a two-item questionnaire (PHQ-2). *Journal of psychosomatic research, 58*(2), 163-171. | PHQ-2 |
| Humeniuk, R., Henry-Edwards, S., Ali, R., Poznyak, V. & Monteiro, M. (2010). *The Alcohol, Smoking and Substance Involvement Screening Test (ASSIST): Manual for use in primary care*. (World Health Organization). | ASSIST 3.1 |
| Bőthe, B., Tóth-Király, I., Demetrovics, Z., & Orosz, G. (2021). The short version of the Problematic Pornography Consumption Scale (PPCS-6): A reliable and valid measure in general and treatment-seeking populations. *The Journal of Sex Research, 58*(3), 342-352.‏ | PPCS-6 |
| Stinchfield, R., Govoni, R. & Frisch, G. R. (2007). A review of screening and assessment instruments for problem and pathological gambling. In Smith, G., Hodgins, D. C. & Williams, R. J (Eds.), *Research and measurement issues in gambling studies* (179–213). Academic Press. | PGSI |
| Lemmens, J. S., Valkenburg, P. M., & Peter, J. (2009). Development and validation of a Game Addiction Scale for adolescents. *Media psychology, 12*(1), 77-95. | GAS |

*Note.* ECR-12 - Experiences in Close Relationships - 12 items, WHOTO - Who To Turn To, MIL - Meaning in Life, CD-RISC - Connor-Davidson Resilience Scale, ECR-RS - Experiences in Close Relationships - Relationship Structures, GAD-2 - Generalized Anxiety Disorder - 2 item screener, PHQ-2 - Patient Health Questionnaire - 2 item screener, ASSIST 3.1 - Alcohol, Smoking and Substance Involvement Screening Test (version 3.1), PPCS-6 - Problematic Pornography Consumption Scale - 6 items, PGSI - Problem Gambling Severity Index, GAS - Gaming Addiction Scale.
